# Resuscitation from hemorrhagic shock after traumatic brain injury with polymerized hemoglobin

**DOI:** 10.1038/s41598-021-81717-3

**Published:** 2021-01-28

**Authors:** Cynthia R. Muller, Vasiliki Courelli, Alfredo Lucas, Alexander T. Williams, Joyce B. Li, Fernando Dos Santos, Clayton T. Cuddington, Savannah R. Moses, Andre F. Palmer, Erik B. Kistler, Pedro Cabrales

**Affiliations:** 1 Department of Bioengineering, University of California San Diego, 9500 Gilman Dr. La Jolla, San Diego, CA 92093-0412 USA; 2 William G. Lowrie Department of Chemical and Biomolecular Engineering, The Ohio State University, Columbus, OH USA; 3 Department of Anesthesiology and Critical Care, University of California San Diego, San Diego, CA USA

**Keywords:** Brain injuries, Trauma

## Abstract

Traumatic brain injury (TBI) is often accompanied by hemorrhage, and treatment of hemorrhagic shock (HS) after TBI is particularly challenging because the two therapeutic treatment strategies for TBI and HS often conflict. Ischemia/reperfusion injury from HS resuscitation can be exaggerated by TBI-induced loss of autoregulation. In HS resuscitation, the goal is to restore lost blood volume, while in the treatment of TBI the priority is focused on maintenance of adequate cerebral perfusion pressure and avoidance of secondary bleeding. In this study, we investigate the responses to resuscitation from severe HS after TBI in rats, using fresh blood, polymerized human hemoglobin (PolyhHb), and lactated Ringer’s (LR). Rats were subjected to TBI by pneumatic controlled cortical impact. Shortly after TBI, HS was induced by blood withdrawal to reduce mean arterial pressure (MAP) to 35–40 mmHg for 90 min before resuscitation. Resuscitation fluids were delivered to restore MAP to ~ 65 mmHg and animals were monitored for 120 min. Increased systolic blood pressure variability (SBPV) confirmed TBI-induced loss of autoregulation. MAP after resuscitation was significantly higher in the blood and PolyhHb groups compared to the LR group. Furthermore, blood and PolyhHb restored diastolic pressure, while this remained depressed for the LR group, indicating a loss of vascular tone. Lactate increased in all groups during HS, and only returned to baseline level in the blood reperfused group. The PolyhHb group possessed lower SBPV compared to LR and blood groups. Finally, sympathetic nervous system (SNS) modulation was higher for the LR group and lower for the PolyhHb group compared to the blood group after reperfusion. In conclusion, our results suggest that PolyhHb could be an alternative to blood for resuscitation from HS after TBI when blood is not available, assuming additional testing demonstrate similar favorable results. PolyhHb restored hemodynamics and oxygen delivery, without the logistical constraints of refrigerated blood.

## Introduction

Hemorrhagic shock (HS) after traumatic brain injury (TBI) increases mortality among military personnel and civilians compared to either of these traumas alone. In the civilian population, more than 26% of TBI patients also present with HS, while the rate is much higher (around 80%) in military personnel, since there is a higher associated incidence of extremity/penetrating injuries^1,2^. Pre-hospital deaths occur in 33–50% of HS after TBI-patients, which suggests that an improved strategy for early intervention/resuscitation might effectively decrease mortality rates in these patients^2^.

Treatment of HS after TBI is particularly challenging as the therapeutic regimens for the two individual conditions may conflict, since ischemia/reperfusion injury associated with resuscitation from HS can be exaggerated due to a loss of local and systemic autoregulatory mechanisms following TBI^3^. Typically, the main therapeutic intervention for HS involves blood transfusion when available, and restrictive use of colloids or crystalloids for fluid resuscitation in order to restore mean arterial pressure (MAP) otherwise^4,5^. However, treatment of TBI aims to maintain adequate cerebral perfusion pressure, cerebral blood flow, and cerebral oxygen (O_2_) delivery without causing cerebral internal bleeding or secondary injury^3,6^. While fluid resuscitation restores perfusion to ischemic tissues and prevents hypoxic and ischemic damage induced by HS, it also has the potential to worsen TBI-related brain pathology, as fluid resuscitation following the loss of local and systemic autoregulatory mechanisms can lead to cerebral edema and diffuse swelling^7,8^. Cerebral edema is one of the most prominent pathophysiological factors associated with death and unfavorable outcomes after TBI^9–11^.

While neurotraumas affect both systemic and cerebrovascular hemodynamic regulatory mechanisms, TBI specifically exacerbates the cardiovascular instability observed during HS^12^. This decompensation manifests as decreased blood pressure regulation via loss of cardiovascular compensatory mechanisms such as control of vascular tone^12–15^. Conversely, the systemic pathophysiological changes attributed to HS exacerbate the inflammatory response induced by TBI, negatively affecting the control of inflammatory mediators and oxidative stress, which in turn affects all organs^16^. Ultimately these pathways contribute to organ failure through microvascular shunting, coagulopathy, blood stasis, capillary occlusion, and acidosis^17,18^. As a result, the optimal treatment goal for HS after TBI is ambiguous, especially since various strategies have been proposed without clear consensus^3^.

Previous studies of combined TBI and HS have indicated that aggressive fluid resuscitation during TBI and HS leads to increased short-term mortality^19^. Consequently, the use of artificial oxygen-carrying blood alternatives, such as hemoglobin (Hb)-based oxygen carriers (HBOCs) could be a possible solution. The use of HBOCs for resuscitation in HS as well as combined TBI and HS has been investigated to a certain degree but conclusions regarding their efficacy remain inconclusive, especially since some of the older generation HBOCs studied caused substantial hypertension, vasoconstriction, and oxidative tissue toxicity^3,20^. A more recently developed large molecular weight polymerized human Hb (PolyhHb), which preserves microvascular hemodynamics and effectively restores O_2_ delivery during HS alone, could be a promising resuscitation alternative to current resuscitation strategies from HS after TBI^21^. Previously, our research group evaluated the efficacy of this next generation PolyhHb in different animal models^22^, but no studies of these therapies on resuscitation from HS after TBI have been completed until now. This study evaluated the efficacy of large molecular weight PolyhHb to restore blood pressure after resuscitation from hemorrhagic shock post-TBI compared to lactated Ringer’s solution (LR, electrolyte solution used to restore the loss of blood volume), and fresh whole blood (Blood, autologous blood drawn during hemorrhage).

## Results

### Systemic hemodynamics

**Figure 1:**
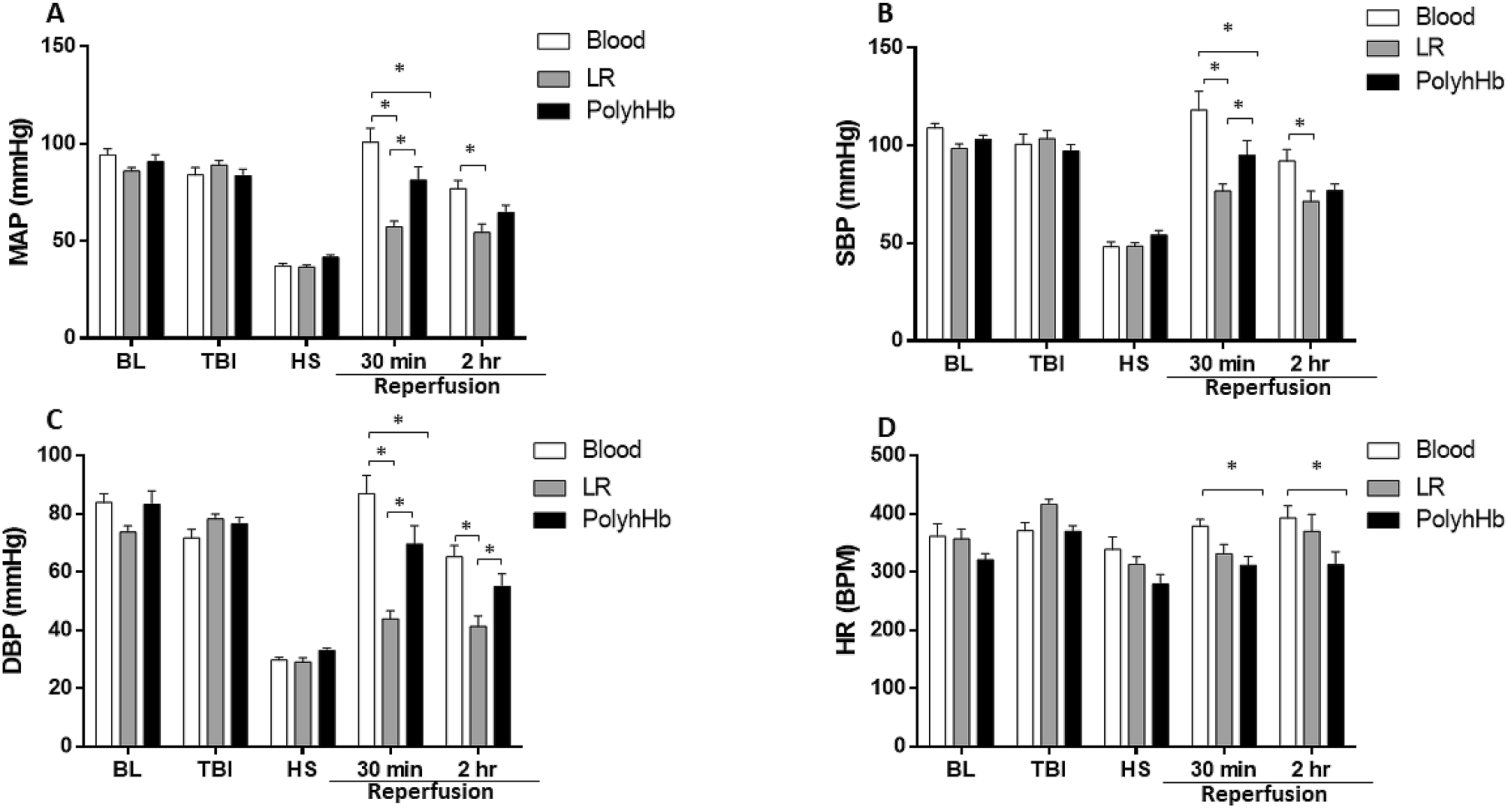
Hemodynamic parameters during the polytrauma protocol. (**A**) Mean arterial pressure (MAP); (**B**) systolic blood pressure (SBP); (**C**) diastolic blood pressure (DBP); (**D**) heart rate (HR). At BL (baseline); TBI (post traumatic brain injury); HS (hemorrhagic shock); 30 min into resuscitation; 2 h into resuscitation. *LR* Lactated Ringer’s solution. *p < 0.05. N = 5/group.

The MAP, systolic blood pressure (SBP), and diastolic blood pressure (DBP) of all groups are presented in Fig. 1A–C, respectively. All blood pressure parameters decreased equally in all groups during HS. The Blood and PolyhHb groups, however, displayed higher MAP compared to the LR group 30 min into resuscitation. Blood and PolyhHb groups were similarly efficient at restoring MAP during resuscitation. The SBP was significantly higher for the Blood and PolyhHb groups compared to the LR group 30 min into resuscitation and remained significantly higher in the Blood group at the end of the resuscitation period. DBP was significantly higher in both Blood and PolyhHb groups compared to the LR group 30 min into resuscitation, and remained significantly higher compared to LR 2 h into resuscitation. No differences were observed in heart rate (HR) between groups at baseline (BL), after TBI, or during HS. HR decreased for all groups during HS (Fig. 1D). After resuscitation, HR increased in the Blood and LR groups but not the PolyhHb group; HR was significantly lower in the PolyhHb group compared to the blood group 30 min into resuscitation, as well as 2 h into resuscitation.

### Spectral blood pressure variability analysis

**Figure 2:**
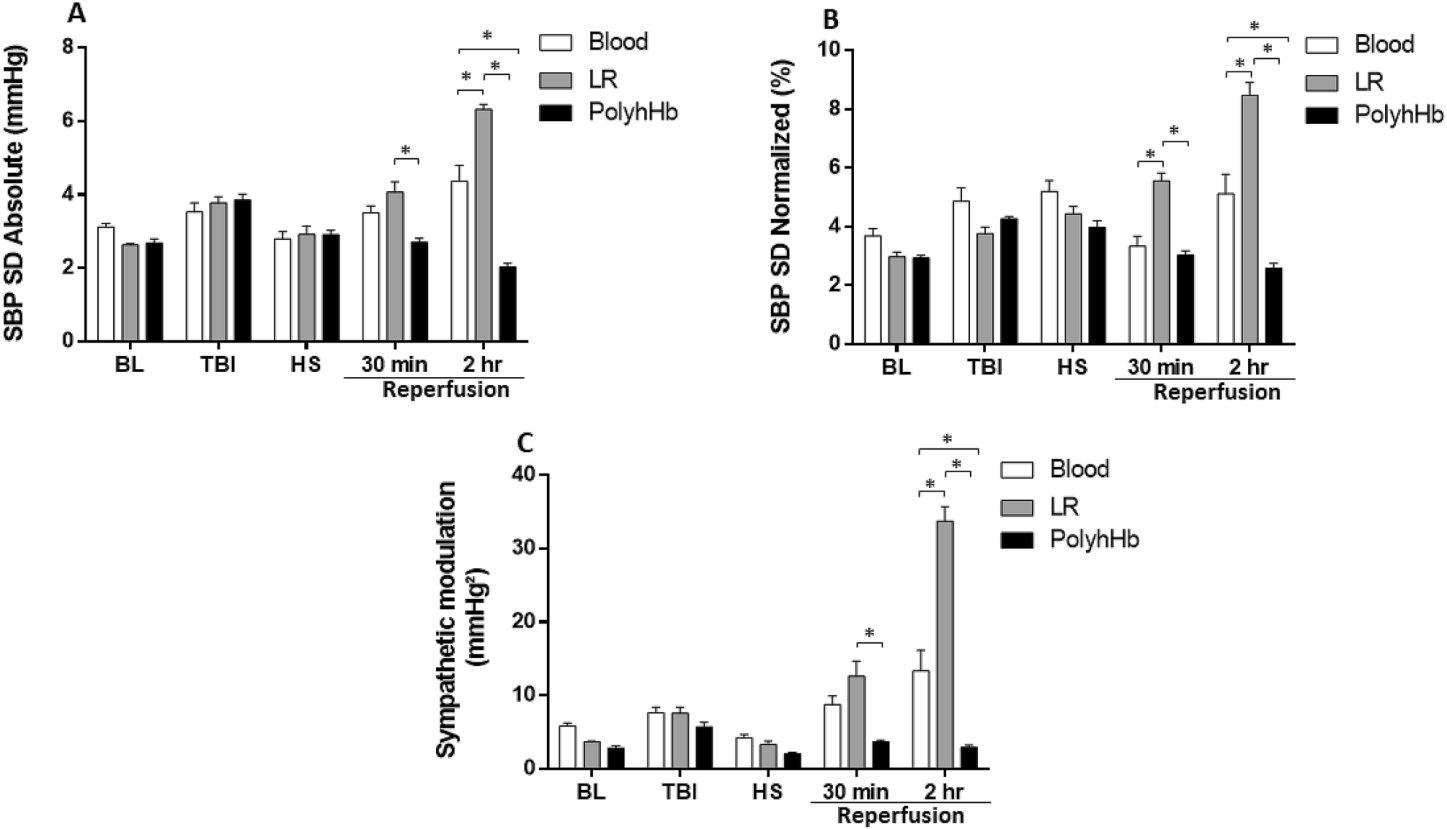
Analysis of systolic blood pressure variability during the polytrauma protocol. (**A**) Systolic blood pressure (SBP) standard deviation (SD) absolute; (**B**) SBP SD normalized; (**C**) sympathetic modulation. At BL (baseline); TBI (post traumatic brain injury); HS (hemorrhagic shock); 30 min into resuscitation; 2 h into resuscitation. *LR* Lactated Ringer’s solution. *p < 0.05. N = 5/group.

The absolute and normalized systolic blood pressure variability (SBPV), and peripheral sympathetic modulation are presented in Fig. 2A–C, respectively. TBI increased SBPV compared to baseline. HS decreased the absolute SBPV compared to TBI, but this decrease was due to a loss of SBP from hemorrhage; when examining relative SBPV, there were no significant differences in relative SBPV between the TBI and HS time points. The PolyhHb group presented decreased SBPV compared to animals resuscitated with LR or blood at the end of the resuscitation period. Consequently, sympathetic modulation was lower in the PolyhHb group compared to the Blood group, and higher for the LR group compared to the Blood group, suggesting increased sympathetic modulation in the LR group and decreased sympathetic modulation in the PolyhHb group (Fig. 2C).

### Blood gases and hematological parameters

Blood gases and hematological parameters are presented in Table 1. Hematocrit decreased in all groups after HS, and only the Blood group recovered hematocrit after resuscitation, as expected. Total hemoglobin (tHb) decreased after HS and was increased following resuscitation in the Blood and PolyhHb groups, but not in the LR group. Arterial O_2_ saturation (SaO_2_) was lower in the Blood and PolyhHb groups compared to the LR group 30 min and 2 h into resuscitation. The SaO_2_ was higher for the Blood group compared to PolyhHb group 2 h into resuscitation. All groups showed an increase in lactate during HS, and 30 min into resuscitation lactate decreased significantly for all groups compared to HS; however, 2 h into resuscitation lactate remained elevated for the PolyhHb group compared to the Blood and LR groups (Table 1).

**Table 1:** Hematological parameters.

	Blood	LR	PolyhHb
**pH**			
BL	7.37 ± 0.01	7.38 ± 0.01	7.40 ± 0.01
HS	7.28 ± 0.03	7.33 ± 0.02	7.36 ± 0.02
Rep 30 min	7.35 ± 0.01	7.35 ± 0.03	7.39 ± 0.02
Rep 2 h	7.41 ± 0.02	7.39 ± 0.03	7.40 ± 0.02
**pO**_**2**_** (mmHg)**			
BL	81.7 ± 1.3	78.9 ± 2.9	95.1 ± 9.5
HS	114.6 ± 3.3	121.5.0 ± 3.5	122.0 ± 3.2
Rep 30 min	84.8 ± 3.0	100.2 ± 3.5	103.5 ± 7.0
Rep 2 h	88.9 ± 2.3	91.2 ± 4.0	106.9 ± 5.7
**pCO**_**2**_** (mmHg)**			
BL	44.4 ± 1.2	46.5 ± 2.0	42.7 ± 0.4
HS	28.3 ± 2.6	30.5 ± 1.8	29.3 ± 1.6
Rep 30 min	40.0 ± 0.9	39.6 ± 0.5	34.5 ± 1.6
Rep 2 h	37.7 ± 1.0	38.4 ± 1.4	32.3 ± 2.3
**HCO**_**3**_** (mmol/L)**			
BL	24.7 ± 0.8	25.8 ± 0.4	25.5 ± 0.6
HS	15.3 ± 1.5	17.6 ± 1.1	18.3 ± 1.2
Rep 30 min	21.6 ± 1.0	21.2 ± 0.4	21.5 ± 1.6
Rep 2 h	24.0 ± 0.9	23.0 ± 0.6	21.1 ± 1.4
**BE (mmol/L)**			
BL	0.2 ± 0.7	2.5 ± 0.5	1.8 ± 0.7
HS	− 13.0 ± 2.3	− 9.7 ± 1.7	− 8.6 ± 1.7
Rep 30 min	− 3.5 ± 1.3	− 4.2 ± 0.5	− 4.1 ± 2.1
Rep 2 h	− 0.6 ± 1.1	− 1.9 ± 0.8	− 4.7 ± 2.0
**O**_**2**_** saturation (%)**			
BL	90.0 ± 0.9	89.1 ± 1.4	90.6 ± 1.2
HS	94.9 ± 0.6	95.8 ± 0.6	96.5 ± 0.4
Rep 30 min	89.9 ± 0.5	95.2 ± 1.0*^†^	87.8 ± 1.5
Rep 2 h	91.8 ± 0.4	98.1 ± 0.7*^†^	87.4 ± 0.6*
**Lactate (mmol/L)**			
BL	1.0 ± 0.1	1.0 ± 0.1	1.0 ± 0.2
HS	7.6 ± 1.1	8.5 ± 1.2	6.2 ± 0.3
Rep 30 min	2.7 ± 0.6	4.5 ± 0.7	4.7 ± 0.5
Rep 2 h	0.9 ± 0.1	1.7 ± 0.3^†^	4.4 ± 0.8*
**Hct (%)**			
BL	43 ± 2	45 ± 1	42 ± 2
HS	33 ± 1	32 ± 1	31 ± 1
Rep 30 min	42 ± 2	27 ± 2*	27 ± 1*
Rep 2 h	40 ± 1	23 ± 1*	25 ± 1*
**tHb (g/dL)**			
BL	13.9 ± 0.6	14.7 ± 0.2	14.2 ± 0.3
HS	10.5 ± 0.4	10.7 ± 0.4	10.2 ± 0.2
Rep 30 min	13.1 ± 0.6	8.4 ± 0.2*^†^	12.4 ± 0.1
Rep 2 h	12.8 ± 0.6	7.0 ± 0.3*^†^	11.9 ± 0.4

Data presented as mean ± SE.

N = 5/group.

*BL* baseline, *HS* hemorrhagic shock, *LR* lactated Ringer’s solution.

*p < 0.05 vs. blood group.

^†^p < 0.05 vs. PolyhHb group.

### Volume requirements

The volume withdrawn to induce and maintain HS was not different between groups (Blood: 9.8 ± 0.8 mL; LR: 9.6 ± 1.2 mL; PolyhHb: 10.4 ± 0.7 mL). For the Blood and PolyhHb groups, the volumes required to achieve the resuscitation goal of 65 mmHg MAP were 6.8 ± 0.8 and 7.5 ± 0.6 mL, respectively and were not significantly different. The volumes given to the Blood and PolyhHb groups during resuscitation represent 69% and 72% of the volumes withdrawn to induce and maintain hypovolemia. No additional volume was required to maintain the goal MAP in these two groups. However, the LR group required a significantly larger resuscitation volume (31.4 ± 3.0 mL) compared to the Blood and PolyhHb groups, representing 336% of the volume required to induce and maintain hypovolemia.

### Inflammation, catecholamines and cortisol

**Figure 3:**
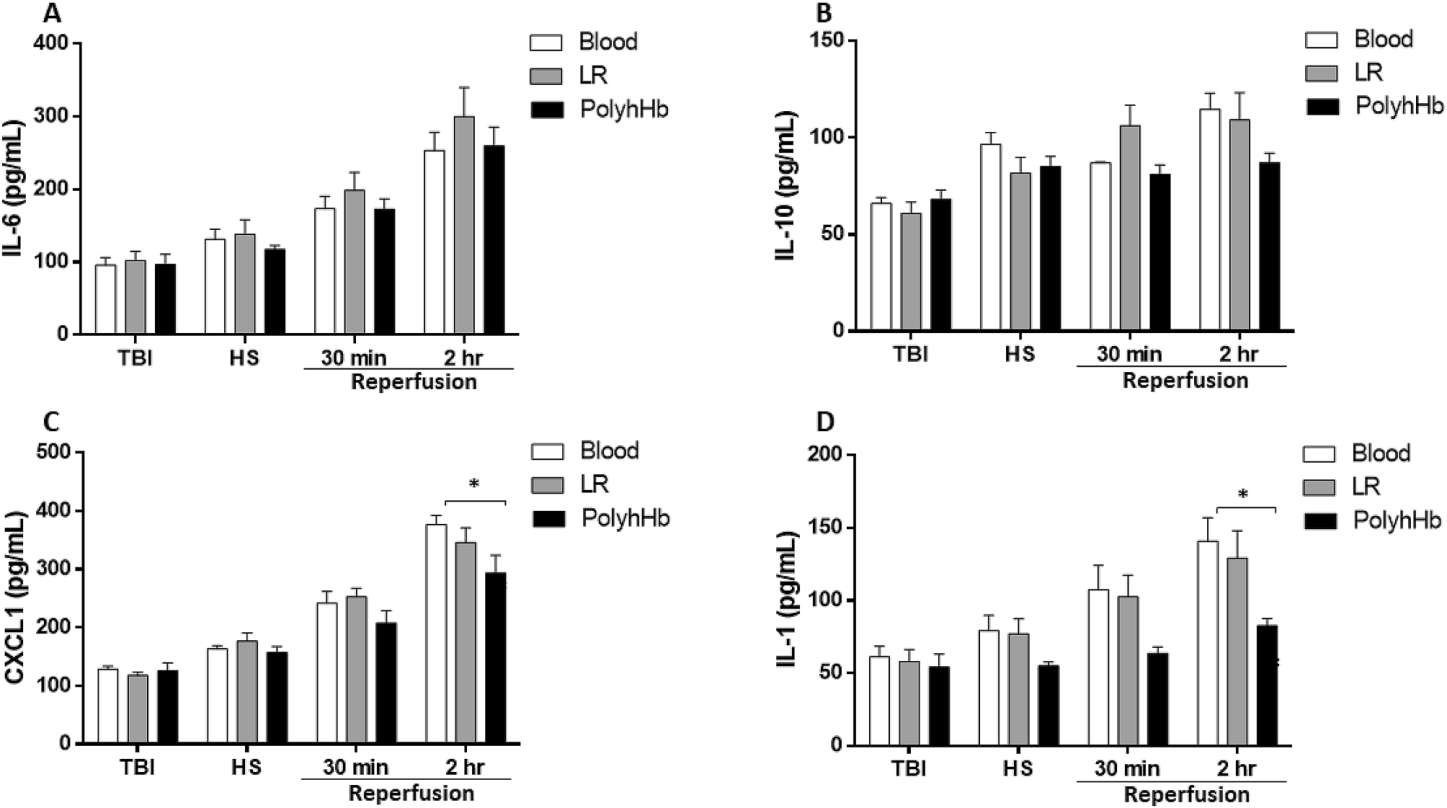
Plasma levels of inflammatory markers. (**A**) Interleukin (IL)-6 (IL-6); (**B**) IL-10; (**C**) Chemokine (CXCL1); (**D**) IL− 1. At BL (baseline); TBI (post traumatic brain injury); HS (hemorrhagic shock); 30 min into resuscitation; 2 h into resuscitation. *LR* Lactated Ringer’s solution. *p < 0.05. N = 5/group.

**Figure 4:**
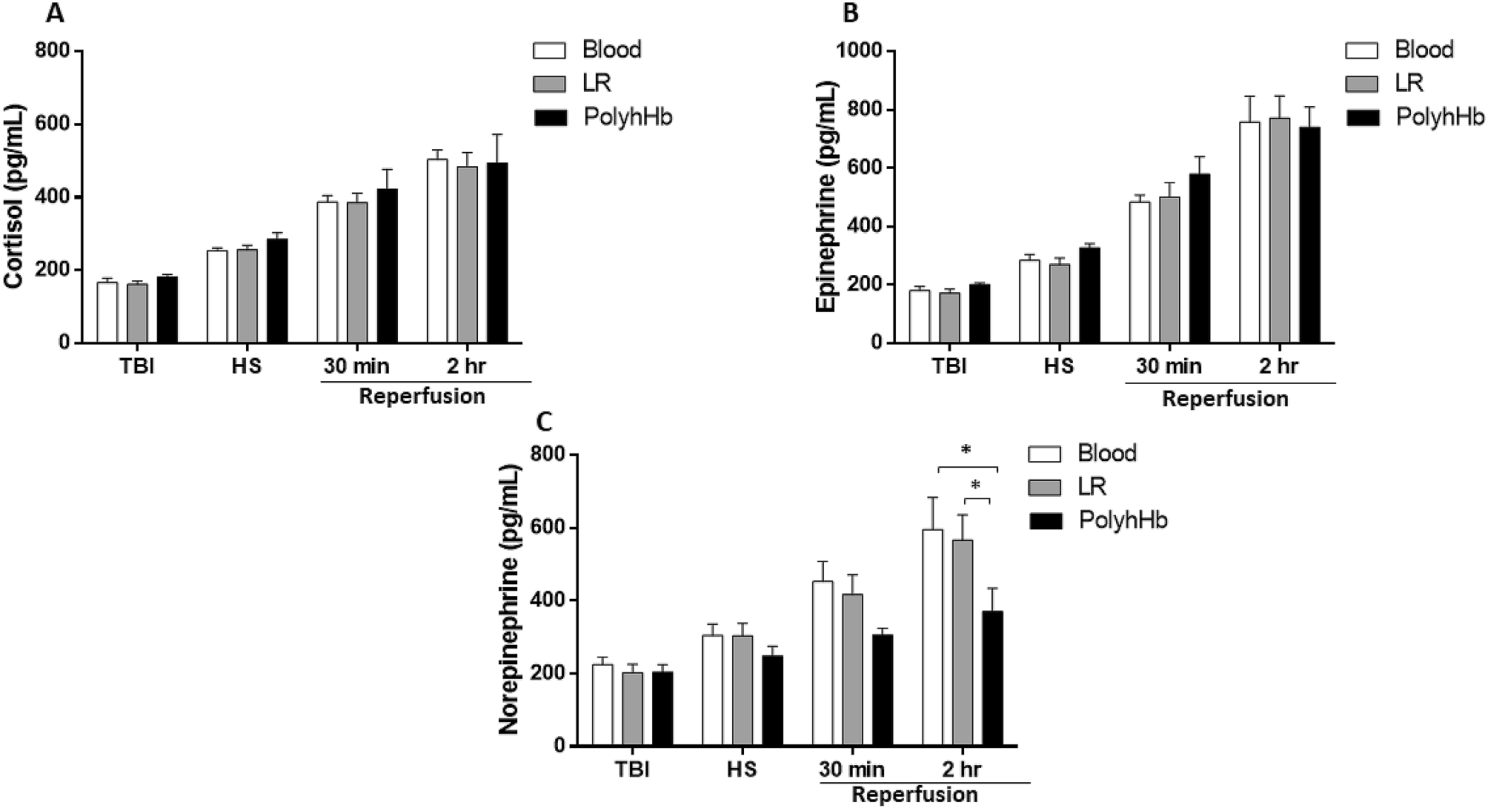
Plasma measurement of cortisol and catecholamines. (**A**) Cortisol; (**B**) epinephrine; (**C**) norepinephrine. At TBI (post traumatic brain injury); HS (hemorrhagic shock); 30 min into resuscitation; 2 h into resuscitation. *LR* Lactated Ringer’s solution. N = 5/group.

In order to determine if PolyhHb activates inflammatory pathways, we measured four classic markers of early inflammation activation: interleukins (IL) 1, 6 and 10, and the chemokine CXCL1. All groups, independent of the resuscitation fluid, showed increased inflammatory markers over the experimental protocol (Fig. 3). No significant differences were observed between groups, but PolyhHb showed slightly lower CXCL1 (Fig. 3C) and IL-1 (Fig. 3D) levels 2 h into the resuscitation period compared to Blood. Catecholamines (epinephrine, norepinephrine) and cortisol (Fig. 4) increased over the experimental protocol, but no statistical differences were observed between groups for cortisol and epinephrine. However, norepinephrine increased less in the PolyhHb group, resulting in lower norepinephrine compared to the Blood and LR groups at the end of the protocol.

## Discussion

PolyhHb has been proposed as an alternative to allogenic blood for use in transfusion medicine due to its’ lack of blood group antigens, reduced immunogenicity, prolonged storage life and stability, tunable oxygen affinity, among other advantageous properties. Here we tested the hypothesis that PolyhHb can restore central hemodynamics during resuscitation from HS after TBI, a first step in evaluating PolyhHb as an alternative to blood when blood is not available or logistic constraints prevent blood transfusion. Resuscitation with PolyhHb restored MAP from HS after TBI to a similar degree as blood. Blood and PolyhHb required significantly less volume for resuscitation compared to LR. The LR group required more than three times the volume withdrawn to restore pressure, but even with the high volume infused, LR was unable to achieve the MAP goal.

Resuscitation from HS after TBI is especially challenging because TBI induces loss of autonomic control, and increases in blood pressure variability. These alterations make it difficult to control and maintain safe pressure levels during resuscitation from HS after TBI. Large resuscitation volumes have been shown to increase cerebral edema and worsen TBI associated sequelae^4^. Resuscitation from HS after TBI with PolyhHb significantly reduced the required volume necessary to restore perfusion pressure compared to resuscitation with LR, with similar volume as whole blood, thus reducing the risk of cerebral edema; unfortunately cerebral edema was not measured in the current study as it requires different instrumentation. The results obtained in this study are promising, but additional studies are necessary to understand the effects of resuscitation with PolyhHb on cerebral hemodynamics, edema, and oxygenation, as well as bleeding and neuropathology, prior to considering clinical use of PolyhHb in HS with TBI.

Increased blood pressure variability (BPV) and peripheral sympathetic modulation post-TBI confirmed loss of autonomic control in our study, and was particularly severe in animals transfused with LR. In contrast, animals transfused with PolyhHb showed decreased blood pressure variability, indicating that PolyhHb could improve resuscitation from HS after TBI by preventing adverse swings in SBP that might lead to or exacerbate intracranial hemorrhage.

Previous studies have shown that PolyhHb increases O_2_ delivery and extraction, and maintains microcirculatory function after resuscitation from HS^23,24^. Based on current and previous studies, non-oxygen carrying crystalloid solutions are ineffective for resuscitation from HS, and this study indicates that they are also ineffective for resuscitation of HS after TBI. The superior recovery of MAP and DBP observed with PolyhHb and blood in this study suggests that O_2_ carrying capacity could be a key factor in restoring hemodynamics during resuscitation from HS after TBI, since the non-oxygen carrier, LR, was not capable of restoring MAP during resuscitation.

Nitric oxide (NO) is a potent vasodilator and its absence could promote vasoconstriction, increased peripheral vascular resistance, and increased MAP during resuscitation from HS. Previous studies have shown that there is an optimal intravascular concentration of NO that improves hemodynamics during resuscitation from HS by restoring pressure, without limiting O_2_ delivery^23^.

Acellular Hb scavenges NO and limits NO availability to the smooth muscle^25^; large molecular diameter HBOCs, like the next generation PolyhHb used in our study, were developed to address this issue and reduce NO scavenging. Although the increased molecular diameter decreases the overall rate of NO scavenging by acellular Hb, we expect that polymerized hemoglobins (PolyHbs) scavenge NO to some extent, which could stabilize MAP when blood pressure variability might otherwise be significantly increased, such as after TBI. The degree of NO scavenging by PolyHbs may not be deleterious in normal circumstances, as our group recently demonstrated that increasing the molecular size of PolyHbs decreases the degree of vasoconstriction, hypertension, and toxicity in a guinea pig model^22^. While NO scavenging by PolyhHb could help increase blood pressure and increase hemodynamic stability, it could also be deleterious by inhibiting protective NO mechanisms, such as reactive hyperemia^26^. Clearly, further studies are necessary to evaluate the implications of NO scavenging by PolyhHb during resuscitation from HS and TBI.

Typically, the sympathoadrenal axis activates in response to blood loss^27^, which acts to vasoconstrict certain vascular beds via neural innervation, resulting in increased sympathetic nervous system (SNS) activity to maintain blood pressure and perfusion in vital tissues, such as the heart, brain, and kidneys. Interestingly, SNS modulation did not change during HS, suggesting that the vasculature was not able to properly adjust its responsiveness when faced with this increase in SNS activity. This effect was likely due to active blood withdrawal through an indwelling catheter, which overcomes the natural vascular contraction response.

SNS modulation increased in animals resuscitated with Blood and LR, but despite this, the LR group was not able to restore MAP. Remarkably, resuscitation with PolyhHb did not demonstrate increased SNS modulation relative to baseline, suggesting that this material restored blood pressure variability and modulation to normal levels by interacting with other mechanisms that promote recovery of MAP. Acute blood loss decreases baroreflex activity, releasing catecholamines into the circulation, which, in addition to neural modulation of blood pressure, acts to restore blood pressure^27^. Circulatory catecholamines increased after HS in all groups and they continued to increase after resuscitation. None of the resuscitation solutions reversed the increase of catecholamines, however, the PolyhHb group showed a smaller increase in norepinephrine levels compared to the other groups, which could impact the PolyhHb group’s recovery of MAP due to norepinephrine’s role in control of vascular tone.

Diastolic blood pressure was restored equally for the groups reperfused with PolyhHb and blood, while LR was unable to restore diastolic pressure. Viscosity is a significant component of vascular resistance, and vascular resistance contributes strongly to maintenance of DBP^28^. As such, the solution viscosities (3.4, 10, and 1 cP for blood, PolyhHb, and LR, respectively) likely contributed to their maintenance (or lack thereof) of DBP. Restoration of DBP is critical, as loss of DBP following resuscitation could exacerbate deterioration of cerebral autoregulation and thus worsen outcomes from HS after TBI.

From our model, the PolyhHb group was able to restore blood pressure and provide hemodynamic stability similar as that achieved with blood transfusion after resuscitation from HS with TBI. During shock, cerebral autoregulation can be disrupted by changes in intracranial pressure or severe blood pressure loss. A phase of decompensation has been shown previously in a model of intracranial pressure increment via the Langfitt curve^29^, where autoregulation stops working and the baroreflex then becomes responsible for maintaining adequate cerebral flow and perfusion^30^. This results in both central and peripheral vasodilation as well as vasoplegia^31^. It is recognized that CO_2_-induced vasodilation in the brain is mediated by the actions of NO^32^. Hence, if PolyhHb scavenges NO in the brain, it could restore vasocontractility and re-establish autoregulation and baroreflex functions, which can explain the better outcomes for blood pressure and peripheral vascular modulation and the shortcomings of the LR and Blood groups. Additional studies on the contribution of oxygen delivery and NO scavenging by PolyhHb need to be conducted to optimize the application of PolyhHb for resuscitation from HS after TBI, as well as to establish any potential detrimental effects of increased oxygenation and reduced NO bioavailability. Large MW PolyhHb has a reduced NO scavenging capacity compared to previous generations of HBOCs^21,22^, however, the implication of limited NO scavenging capability of PolyhHb needs to be studied in the context of TBI with HS in future studies.

TBI patients often exhibit multiple injuries and frequently present with concomitant HS, increasing the risk for developing severe sustained systemic inflammation and immunological dysfunction. In turn, the immunologic and inflammatory response resulting from HS likely exacerbates the severity of TBI. TBI disrupts the blood–brain barrier and other protective mechanisms of the brain, exposing the brain to significantly higher levels of both systemic inflammatory markers and markers of peripheral immune response compared to HS alone^33^. We observed increased systemic inflammatory markers during the shock period, and inflammatory markers were not ameliorated with any of the resuscitation solutions. This inflammation may contribute to the severity of TBI in our experimental model.

TBI significantly shifts metabolism, and causes organisms to preferentially utilize lactate instead of glucose^34^. Unpublished results from our group using the same HS model (without TBI), showed that glucose blood levels decrease during HS, and it was necessary to supplement rats with additional glucose in order to prevent death before the end of the resuscitation period. However, in this study supplemental glucose was not needed, suggesting that the TBI indeed led to the use of lactate as a preferential energy source. Interestingly, lactate remained higher for the PolyhHb group than the other groups. We hypothesize that this is due to the fact that PolyhHb is suspended in LR.

A recently published study from our group showed that O_2_ delivery, solution viscosity, and colloid osmotic pressure (COP) appear to be more important than the total volume infused during fluid resuscitation for recovering blood pressure, cardiac function, and blood flow^35^. The PolyhHb tested in this study exhibits higher viscosity and oxygen carrying capacity than crystalloid solutions. Taken together, these characteristics of PolyhHb result in a solution that effectively restores MAP and specifically DBP in a severe model of HS and TBI. Other HBOC formulations have been shown to protect the brain from secondary neurodegeneration in similar models of HS and TBI, indicating that HBOC transfusion could be a strategy to improve outcomes during resuscitation from HS after TBI^36^.

### Limitations

The present study was conducted in anesthetized animals, and isoflurane has potent dose-dependent cardiovascular and cerebral effects, causing myocardial depression, vasodilation, and HR depression, potentially mitigating the dynamics of HR variability measurements. Isoflurane also induces cerebral vasodilation, increased cerebral blood flow, decreased cerebral metabolism, and impaired cerebral blood flow autoregulation^37^. However, the implication of isoflurane to the results of the current study were equal for all experimental groups as all animals were subjected to an equal dose of isoflurane during the protocol, to mitigate the effects of isoflurane in determining the outcomes of the study. Additionally, the impact of PolyhHb on expression of inflammatory markers may not be observed during the time span of the experiment; thus, further studies are needed to better understand the systemic inflammatory response to these fluids after resuscitation from HS after TBI.

Another important limitation is that TBI with HS is known to lead to complex coagulopathy, and the severity of the coagulopathy can be exacerbated by dilutional coagulopathy with non-hemostatic agents. Since PolyhHb possesses low colloidal osmotic pressure (< 10 mmHg)^35^, it does not induce volume expansion beyond the volume transfused, reducing the severity of dilutional coagulopathies relative to colloid fluids (such as hydroxyethyl starch or gelatins), it also requires less volume than crystalloids to restore hemodynamic parameters, such as that observed with LR in this study. To clearly assess the hemostatic implications of PolyhHb after resuscitation from HS with TBI, specific studies need to be implemented in the future.

Additionally, intravenous administration of heparin could influence the polytrauma associated pathology, either by preventing clot formation and subsequent thrombolysis, or by increasing TBI induced bleeding. Unexpected bleeding was not observed during the study, and all animals were monitored for any sign of bleeding. On the other hand, further studies are clearly necessary to examine the effects of PolyhHb on coagulation and cerebral microvascular blood flow during resuscitation from HS after TBI. Finally, we did not include a non-resuscitation group, as previous observations suggest that the protocol used is lethal when no resuscitation fluid is used.

In conclusion, our results show that PolyhHb was effective in resuscitating animals after experimental HS with TBI, restoring blood pressure and pressure stability to a similar extent as resuscitation with blood, and with a lower required volume and superior hemodynamic stability compared to LR. Based on these initial studies, PolyhHb should be further explored as an alternative to blood transfusion for field resuscitation from HS after TBI, particularly in environments where blood is not available (such as emergency situations, and on the battlefield). Although these results are promising, further studies are necessary to generate a complete understanding of the effects of resuscitation with PolyhHb within the cerebral microenvironment after resuscitation from hemorrhage post TBI. Additionally, further studies should include larger animal models to improve translation applicability of these results.

## Methods

### Animal preparation

Studies were performed in male Wistar rats weighing 350–400 g (Charles River Laboratories, Wilmington, MA). Animal handling and care followed the NIH Guide for Care and Use of Laboratory Animals, and all protocols were approved by the University of California San Diego Institutional Animal Care and Use Committee. All methods were carried out in accordance with the ARRIVE guidelines (Animal Research: Reporting of In Vivo Experiments). Briefly, animals were anesthetized using isoflurane 5% in compressed room air for induction (Drägerwerk AG, Lübeck, Germany) and then maintained at 1.5%, except during surgical procedures when it was briefly increased to 2.5%. Animals were placed on a heating pad to preserve core body temperature at 37 °C and allowed to freely breathe from a nosecone delivering anesthesia. Animals were instrumented with a right femoral artery and vein catheter for hemodynamic assessment, and blood withdrawal and intravenous infusion, respectively. Animals were allowed to stabilize for 10 min and baseline measurements were collected.

### Traumatic brain injury

**Figure 5:**
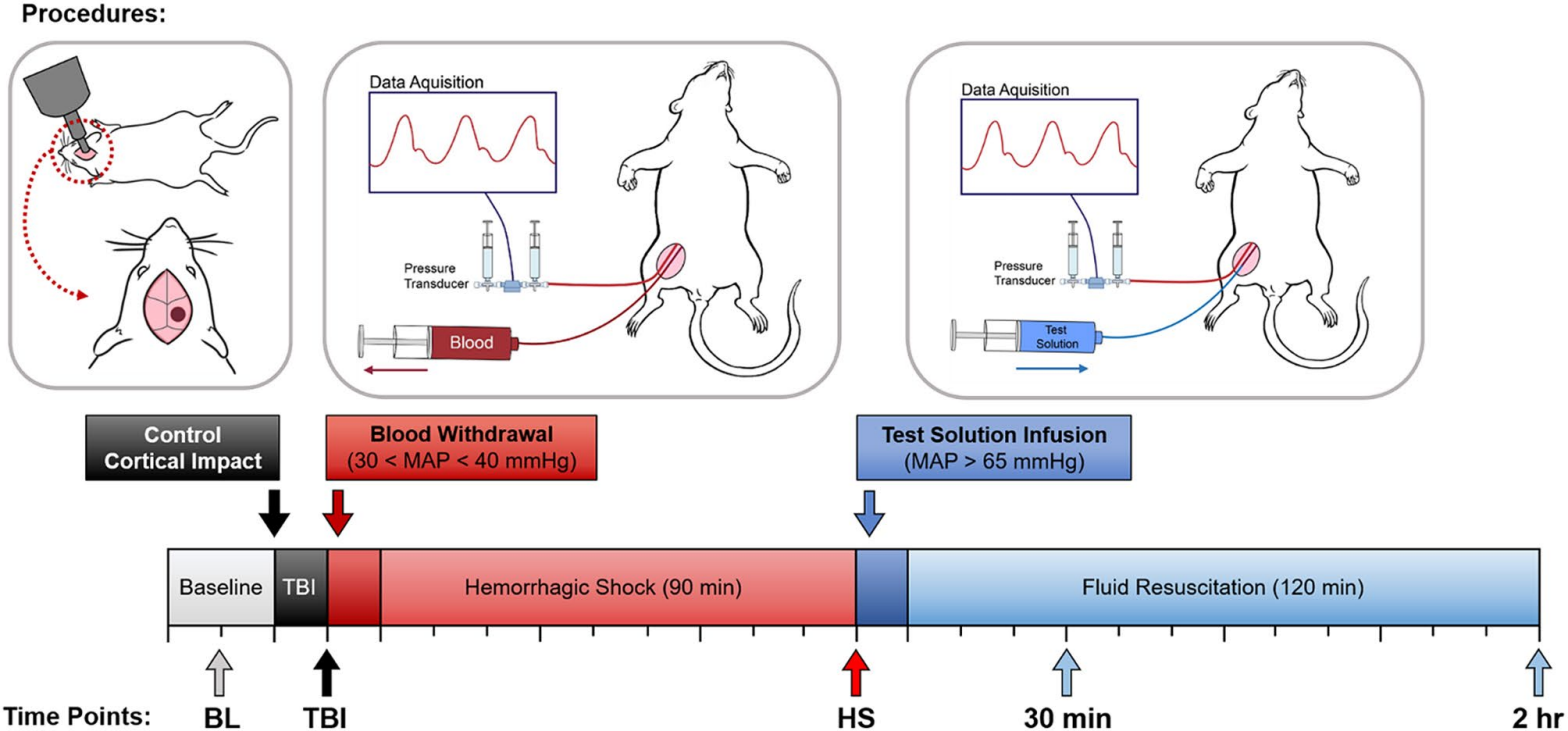
Representative timeline for the experimental protocol.

Animals were then moved to a stereotaxic apparatus and placed in the ventral position, and the isoflurane was increased to 2.5% for 5 min before inducing TBI. To induce TBI, a 5 mm craniotomy was performed over the right cerebral cortex, and the dura was impacted with a 5.0 mm flat tipped impactor at a velocity of 5 m/s and dwell time of 200 ms via a pneumatically controlled cortical impactor (CCI; Leica Biosystems, Vista, CA). After CCI, the head was closed and the animals were placed back on the heating pad dorsally, and the isoflurane was decreased to 1.5%/vol for 10 min before starting hemorrhagic shock (HS) (Fig. 5).

### Hemorrhagic shock

All animals were given 10 min to stabilize after performing the TBI before performing HS. Animals were intravenously heparinized (100 IU/kg) to ensure patency of the catheters during the protocol. Hemorrhage was induced by removing blood from the femoral vein (0.5 mL/min) until the MAP reached 40 mmHg. The MAP was maintained between 35 and 40 mmHg for 90 min by withdrawing or returning small volumes of blood when MAP was out of the indicated range for more than 2 min. After the prolonged severe hypovolemia, animals were randomly assigned to one of three groups (n = 5/group) based on the test intravenous resuscitation fluid: lactated Ringer’s (LR, Baxter Health Care Corporation, USA), blood collected during the hemorrhage (total Hb concentration: approximately 11 g/dL), or PolyhHb (total Hb concentration: 10 g/dL). All resuscitation solutions were administered intravenously at 2 mL/min until the MAP reached 65 mmHg or greater, and additional fluid was intravenously infused to maintain MAP at 65 mmHg when MAP dropped from the target pressure for more than 2 min. Animals were monitored for 120 min from the beginning of the resuscitation until euthanasia. Blood samples were taken at baseline (BL), 90 min into HS (HS), and 30 min and 2 h after resuscitation. After samples were collected at the final time point, animals were euthanized via a single IV injection of sodium pentobarbital (300 mg/kg) (“Supplementary Information S1”).

### Systemic parameters

Arterial pressure and heart rate (HR) were recorded continuously from the femoral artery (MP150, Biopac, Santa Barbara, CA) at a 2 kHz sampling rate. Blood pressure recordings were used to calculate online mean arterial pressure (MAP), systolic blood pressure (SBP), and diastolic blood pressure (DBP) using AcqKnowledge software (Biopac). Hematocrit was measured from centrifuged arterial blood samples taken in heparinized capillary tubes. Arterial and venous blood was collected in heparinized glass capillary tubes (65 µL) and immediately analyzed for oxygen partial pressure (PO_2_), carbon dioxide partial pressure (PCO_2_), pH, Hb saturation, glucose, and lactate (ABL90; Radiometer America, Brea, CA).

### Inclusion criteria

Animals were suitable for experiments if: (1) mean arterial blood pressure (MAP) was greater than 85 mmHg at baseline, (2) lactate less than 1.5 mmol/L at baseline, (3) systemic Hb was greater than 12 g/dL at baseline, and (4) animals survived the hypovolemic period.

### Polymerized human hemoglobin

PolyhHb was synthesized in the low O_2_ affinity tense quaternary state (T-state) at a 30:1 molar ratio of glutaraldehyde to human Hb, and then subjected to 8–9 cycles of diafiltration on a 500 kDa hollow fiber filter. This resulted in a PolyhHb solution containing only polymerized Hb molecules with MW greater than 500 kDa but less than 0.2 µm in size. PolyhHb was produced and characterized at The Ohio State University and shipped overnight frozen to UC San Diego where it was stored at − 80 °C until use. The preparation and characterization of PolyhHb has been previously described in the literature^38,39^.

### Inflammatory markers, catecholamines, and cortisol

Blood (0.5 mL) was collected in 1.5 mL Eppendorf tubes and immediately centrifuged, plasma collected and stored at − 80 °C for future measurements. Inflammatory markers IL6 (BMS625), IL10 (BMS629), IL1(RLB00, R&D Systems, Minneapolis, MN), CXCL-1 (ERCXCL1) (Thermo Fisher, Waltham, MA), catecholamine (BA-E-6600 ImmunoSmol, France) and cortisol (CSB-E05112r, Cusabio, TX) analyses were performed using enzyme-linked immunosorbent assay (ELISA) kits according to the manufacturer’s instructions.

### Spectral blood pressure variability (SBPV)

Temporal series of systolic blood pressure (SBP) were analyzed in the time domain, obtaining the absolute variability of SBP (SBP SD Absolute) by standard deviation every 10 min. Due to different blood pressure levels over time, the variability was also normalized by SBP at each time point and presented as a percentage of variation (SBP SD Normalized). For the frequency domain, the interpolated waves were divided into segments of 512 points, with an overlap of 50%, and were processed by Fast Fourier Transform. One spectrum was obtained for each segment and the oscillatory components were quantified in the frequency domain from 0.20 to 0.75 Hz, representing peripheral sympathetic modulation. The frequency domain response is presented as absolute modulation values (mmHg^2^) averaged over a 10 min period (Sympathetic Modulation).

### Statistical analysis

All values are expressed as mean ± SE. Data were analyzed using Two-Way Analysis of Variance (ANOVA) for repeated measurements. A priori sample size calculation was based on power analysis using preliminary results from a pilot study and produced an estimated group sample size of five animals per group. As data was collected during the study, power analysis was implemented to confirm the appropriate number of animals per group sufficient to provide power for the study (1 − β > 0.8). When appropriate, post hoc analyses were performed with the Tukey multiple comparisons test. All statistics were calculated using GraphPad Prism 6 (GraphPad Software, Inc., San Diego, CA). Results were considered significant if p < 0.05.

## Supplementary Information

Supplementary Figures.
