# Supplementary material for: Resuscitation from hemorrhagic shock after traumatic brain injury with polymerized hemoglobin
Source: Sci Rep. 2021 Jan 28;11:2509. doi: 10.1038/s41598-021-81717-3 (PMC7843604; doi:10.1038/s41598-021-81717-3)

**Resuscitation from Hemorrhagic Shock after Traumatic Brain Injury with Polymerized Hemoglobin**

Cynthia R. Muller^1^, Vasiliki Courelli^1^, Alfredo Lucas^1^, Alexander T. Williams^1^, Joyce B. Li^1^, Fernando Dos Santos^3^, Clayton T. Cuddington^2^, Savannah R. Moses^2^, Andre F. Palmer^2^, Erik B. Kistler^3^, Pedro Cabrales^1^*


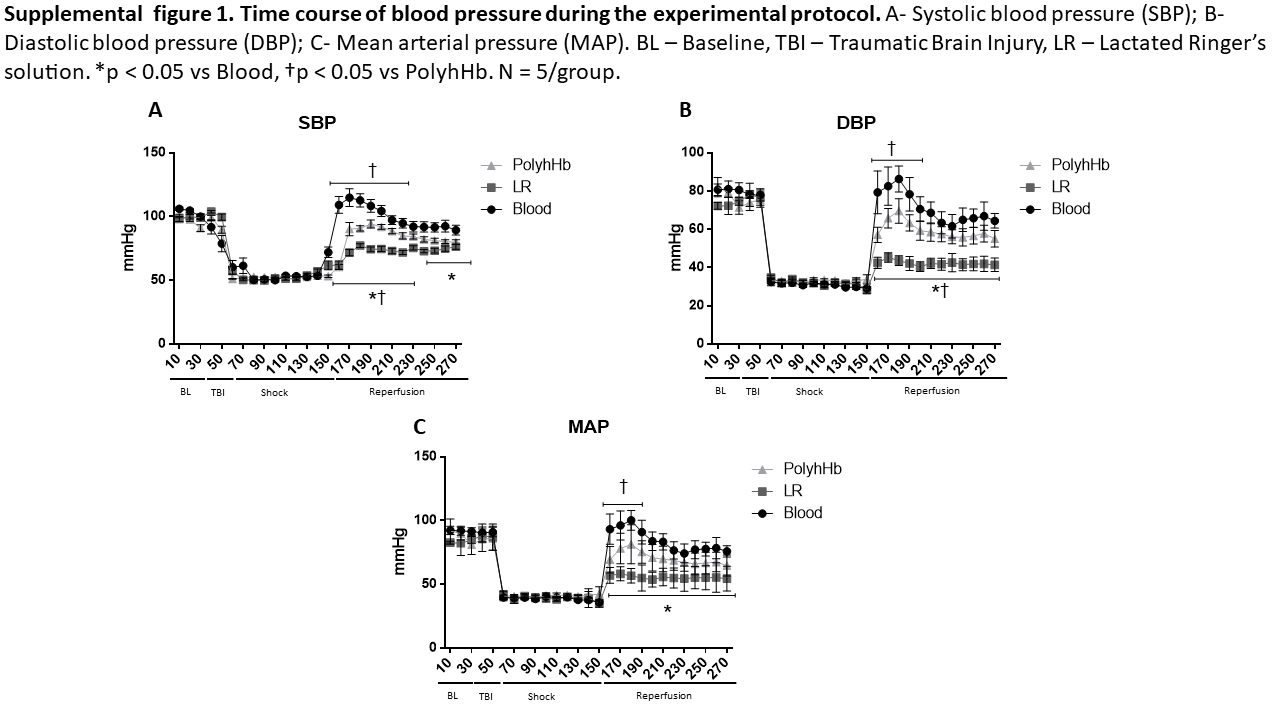


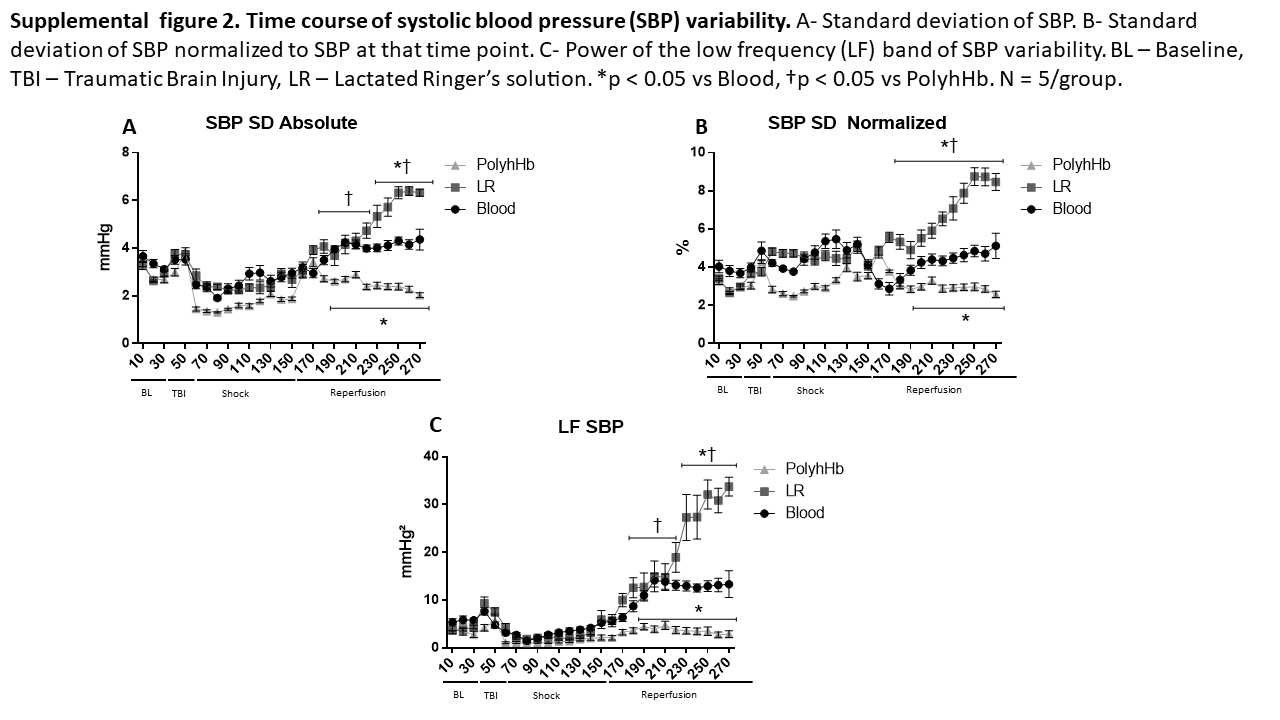

Supplement: Supplementary file 1 — Supplementary Figures. [file 41598_2021_81717_MOESM1_ESM.docx]
